# Tumor Necrosis Factor-Alpha and Interleukin-6 Gene Polymorphisms in Iranian Patients with Ischemic Heart Failure

**Published:** 2018

**Authors:** Mona Hedayat, Mohammad Jafar Mahmoudi, Mohammad Taghvaei, Ebrahim Nematipour, Elham Farhadi, Nilufar Esfahanian, Maryam Mahmoudi, Maryam Sadr, Keramat Nourijelyani, Ali Akbar Amirzargar, Nima Rezaei

**Affiliations:** 1. Division of Immunology, Boston Children’s Hospital, Harvard Medical School, Boston, MA, USA; 2. Network of Immunity in Infection, Malignancy and Autoimmunity (NIIMA), Universal Scientific Education and Research Network (USERN), Boston, MA, USA; 3. Division of Cardiology, Department of Internal Medicine, School of Medicine, Tehran University of Medical Sciences, Tehran, Iran; 4. Molecular Immunology Research Center, Tehran University of Medical Sciences, Tehran, Iran; 5. Tehran Heart Center, Tehran University of Medical Sciences, Tehran, Iran; 6. Hematology Department, Faculty of Allied Medical Science, Iran University of Medical Sciences, Tehran, Iran; 7. School of Nutrition and Dietetics, Tehran University of Medical Sciences, Tehran, Iran; 8. Dietitian and Nutrition Experts Team (DiNET), Universal Scientific Education and Research Network (USERN), Tehran, Iran; 9. Department of Epidemiology and Biostatistics, School of Public Health, Tehran University of Medical Sciences, Tehran, Iran; 10. Research Center for Immunodeficiencies, Children’s Medical Center, Tehran University of Medical Sciences, Tehran, Iran; 11. Department of Immunology, Faculty of Medicine, Tehran University of Medical Sciences, Tehran, Iran; 12. Medical Genetics Network (MeGeNe), Universal Scientific Education and Research Network (USERN), Tehran, Iran

**Keywords:** Genes, Heart failure, Interleukin-6, Tumor necrosis factor-alpha

## Abstract

**Background:**

Proinflammatory cytokines have been known to be elevated in patients with Chronic Heart Failure (CHF). Given the importance of proinflammatory cytokines in the context of the failing heart, the prevalence of Tumor Necrosis Factor-α (TNF-α), Interleukin (IL)-6 polymorphisms in patients with CHF was studied due to ischemic heart disease.

**Methods:**

Forty three patients with ischemic heart failure were enrolled in this study and compared with 140 healthy individuals. The allele and genotype frequency of four Single Nucleotide Polymorphisms (SNPs) within the IL-6 (-174, nt565) and TNF-α (-308, -238) genes were determined, using Polymerase Chain Reaction with Sequence-Specific Primers (PCR-SSP) assay.

**Results:**

The frequency of the TNF-α (-238) A/A genotype was significantly higher in patients comparing to controls (p=0.043), while TNF-α G/A genotype at the same position decreased significantly, in comparison with controls (p=0.018). The most frequent haplotype for TNF-α was A/A in the patient group in comparison with controls (p=0.003). There was no significant difference in allele and genotype frequencies of IL-6 at positions -174 and nt565, and TNF-α at position -308.

**Conclusion:**

Certain alleles, genotypes, and haplotypes in TNF-α, but not IL-6, gene were overrepresented in patients with ischemic heart failure, which may, in turn, predispose individuals to this disease.

## Introduction

Chronic Heart Failure (CHF) is among the leading causes of mortality worldwide, with an incidence rate of 10 per 1000 population after the age of 65^1,2^. Given the increasing incidence of the disease, identifying groups of patients who may be genetically more susceptible to developing CHF would be essential. Thus initiating therapy at an early stage of the disease could be considered.

Increased levels of proinflammatory cytokines, including Tumor Necrosis Factor-α (TNF-α) and Interleukin (IL)-6, have been previously reported in patients with CHF^3–6^. Meanwhile, their roles in the development and progression of the underlying Ischemic Heart Disease (IHD) are also well established^7,8^. Genetic polymorphisms within the coding and promoter regions of proinflammatory cytokine genes have been described to regulate gene expression, ultimately altering cytokine production^9–11^. The association between some cytokine gene polymorphisms and a number of diseases has been previously studied^12–17^. Indeed association of TNF-α with the susceptibility to and severity of CHF has also been examined. The promoter region of TNF-α gene contains several SNPs which influence gene expression or cytokine secretion. In addition to genetic variations, environmental stimuli of inflammatory processes, such as smoking and obesity, were shown to influence TNF-α protein concentrations, further contributing to individualized differences in TNF-α level^18,19^. The overall contribution of genetic variation to the development of CHF is not well established. TNF-α -308G/A polymorphism is one of the widely studied polymorphisms in CHF; however, despite their association with other inflammatory diseases, a comprehensive review of the literatures fails to show any relationship between TNF-α polymorphism and the presence of CHF or the elevation of circulating TNF-α^20^. However, the role of polymorphisms in other proinflammatory cytokine genes in CHF has not been fully investigated.

The objective of this research was to study proinflammatory cytokine gene polymorphisms in Iranian patients with CHF due to IHD.

## Materials and Methods

### Subjects

In the present study, a total of 43 consecutive Iranian patients with chronic ischemic heart failure (mean age 60.05±11.97 yr; 34 men, 9 women) with angiographically significant Coronary Artery Disease (CAD), defined as ≥50% diameter stenosis in at least one of the major coronary arteries, were enrolled. The diagnosis of CHF was based on impaired left ventricular systolic function (left ventricular ejection fraction ≤40%) and left ventricular dilation (left ventricular end-diastolic diameter >5.5 *cm*) on echocardiography. All patients underwent transthoracic echocardiography and cardiac catheterization. Subjects with chronic lung disease, malignancies, recent myocardial infarction, and acute decompensated heart failure within 3 months before recruitment were excluded. Eligible patients were in stable clinical condition and received conventional medical therapy for at least 3 months.

One hundred and forty healthy control subjects were randomly selected from blood donors at Iranian blood transfusion organizations, as previously described^21^. Written informed consent was obtained from all participants prior to blood sampling. This study was approved by the Ethical Committee of Tehran University of Medical Sciences.

### Genotyping

Genomic DNA was isolated from peripheral blood leukocytes, using saltingout method. Cytokine typing was performed by polymerase chain reaction with sequence-specific primers (PCR-SSP) assay (PCR-SSP kit, Heidelberg University, Heidelberg, Germany), as previously described in details^21^. Briefly, amplification was carried out using a thermal cycler Techne Flexigene apparatus (Rosche, Cambridge, UK). The presence or absence of PCR products was visualized by 2% agarose gel electrophoresis. All individuals were genotyped for polymorphic sites of the following cytokine genes: IL-6, -174 G/C and nt565 G/A; TNF-α, -308 G/A and -238 G/A.

### Statistical analysis

Statistical analyses were performed with EPI info software. Allele, genotype, and haplotype frequencies for all cytokine gene polymorphisms were calculated by direct counting. Frequencies of alleles, genotypes, and haplotypes were compared between the patient and control groups using the Fisher’s exact test. The odds ratio with 95% confidence intervals was calculated.

## Results

### Alleles and genotype frequencies

Allelic and genotype frequencies in patients with ischemic heart failure and healthy controls are presented in table 1. It should be noted that among 140 controls enrolled in the original study^21^, results of three controls were not conclusive for TNF-α, while no result was detected for IL-6 of a control.

**Table 1.** Comparisons of allele and genotype frequencies between patients with ischemic heart failure and controls

**Cytokine**	**Position**	**Alleles/Genotypes**	**Patients (n=43)** **N (%)**	**Controls (n=140)** **N (%)**	**p-value**	**Odds ratio (95% CI)**
**TNF-α**						
	-308	A	14 (16.3)	39 (14.2)	0.606	1.17 (0.60 to 2.28)
		G	72 (83.7)	235 (85.8)	0.606	0.85 (0.44 to 1.66)
		AA	1 (2.3)	0 (0)	0.239	9.71 (0.39 to 242.9)
		GA	12 (27.9)	39 (28.5)	1.000	0.97 (0.45 to 2.09)
		GG	30 (69.8)	98 (71.5)	0.848	0.92 (0.43 to 1.94)
	-238	A	15 (17.4)	59 (21.5)	0.449	0.77 (0.41 to 1.44)
		G	71 (82.6)	215 (78.5)	0.449	1.30 (0.69 to 2.43)
		AA	3 (7)	1 (0.7)	0.043*	10.20 (1.03 to 100.8)
		GA	9 (20.9)	57 (41.6)	0.018*	0.37 (0.17 to 0.83)
		GG	31 (72.1)	79 (57.7)	0.108	1.90 (0.90 to 4.01)
**IL-6**						
	−174	C	26 (30.2)	101 (36.3)	0.365	0.76 (0.45 to 1.28)
		G	60 (69.8)	177 (63.7)	0.365	1.32 (0.78 to 2.22)
		CC	2 (4.6)	4 (2.9)	0.628	1.65 (0.29 to 9.32)
		GC	22 (51.2)	93 (66.9)	0.072	0.52 (0.26 to 1.04)
		GG	19 (44.2)	42 (30.2)	0.099	1.83 (0.91 to 3.69)
	+565	A	16 (18.6)	50 (18)	0.874	1.04 (0.56 to 1.944)
		G	70 (81.4)	228 (82)	0.874	0.96 (0.51 to 1.79)
		AA	0 (0)	4 (2.9)	0.574	0.35 (0.018 to 6.56)
		GA	16 (37.2)	42 (30.2)	0.455	1.37 (0.67 to 2.80)
		GG	27 (62.8)	93 (66.9)	0.713	0.835 (0.41 to 1.70)

A significant positive association with the A/A genotype was found for TNF-α at position -238 in our patients compared to controls (7 *vs*. 0.7%, p=0.043), while TNF-α G/A genotype at the same position decreased significantly in patients compared to controls (20.9 *vs*. 41.6%, p=0.018).

The allele and genotype frequencies of IL-6 at positions -174 and nt565, and TNF-α at position -308 were similar in two groups of patients and controls.

### Haplotype frequencies

Haplotype frequencies in patients with ischemic heart failure and healthy control subjects are shown in table 2. The most frequent haplotype for TNF-α (positions -308, -238) was A/A in the patient group in comparison with controls (4.7 *vs*. 0%, p=0.003).

**Table 2.** Comparisons of haplotype frequencies of TNF-α and IL-6 between patients with ischemic heart failure and controls

**Cytokine**	**Haplotype**	**Patients (n=43)** **N (%)**	**Controls (n=140)** **N (%)**	**Odds ratio (95% CI)**	**p-value**
**TNF-α (-308, -238)**	GG	61 (70.9)	176 (64.2)	1.36 (0.80 to 2.30)	0.298
	AG	10 (11.6)	39 (14.2)	0.79 (0.38 to 1.66)	0.594
	GA	11 (12.8)	59 (21.5)	0.53 (0.27 to 1.07)	0.086
	AA	4 (4.7)	0 (0)	29.95 (1.6 to 562.4)	0.003^*^

**IL-6 (-174, nt565)**	GG	57 (66.3)	173 (62.2)	1.19 (0.72 to 1.98)	0.525
	CG	13 (15.1)	55 (19.8)	0.72 (0.37 to 1.4)	0.429
	CA	13 (15.1)	46 (16.6)	0.90 (0.46 to 1.76)	0.868
	GA	3 (3.5)	4 (1.4)	2.51 (0.55 to 11.45)	0.361

## Discussion

There is a paucity of data in the literature on the association of TNF-α -238G/A polymorphism with CHF. Bruggink *et al* reported increased frequency of TNF-α -238/A allele in patients suffering from dilated cardiomyopathy on left ventricular assisted device compared to patients on medical therapy^22^. However, no such association was reported in patients with ischemic heart failure^22^. In the present study, the TNF-α (-238) A/A genotype frequency in patients with ischemic heart failure was significantly higher, while the G/A genotype at the same position was significantly lower than controls. Moreover, the frequency of TNF-α (-308, -238) A/A haplotype was higher in patients compared to controls. To the best of our knowledge, this is the first report describing an association between TNF-α (-238) A/A genotype and TNF-α (-308, -238) A/A haplotype with ischemic heart failure. The number of patients with this haplotype was low; however, it should be emphasized that a very rare haplotype was found that was more common among patients with ischemic heart failure than healthy controls. Although this might be a chance finding, the results need to be replicated in other, preferably larger, population with greater haplotype diversity. Moreover, given the small number of patients in this study, any conclusions can only be interpreted with caution.

There are conflicting reports on the influence of TNF-α -238G/A polymorphism on the expression level of TNF-α. The TNF-α -238/A allele has been reported to be associated with both increased and decreased TNF-α expression^9,23^, whilst other investigators reported no significant association between TNF-α -238G/A polymorphism and the cytokine expression level^24,25^. A single SNP, together with other SNPs, may only be able to modify the disease phenotype in an appropriate environmental context. Therefore, circulating TNF-α levels might reflect combined effects of multiple SNPs, in addition to environmental factors. This might in part explain the conflicting reports regarding the influence of TNF-α -238G/A polymorphism on the expression level of TNF-α.

Increased levels of IL-6 have been described repeatedly in patients with CHF with a positive correlation with disease severity. Previous studies have also reported the association between the polymorphisms in the promoter region of the IL-6 gene and altered cytokine production^10,26^. However, to the best of our knowledge, the association between IL-6 gene polymorphisms and risk of ischemic heart failure has not been reported previously. In the present study, no association between -174G/C and +565A/G polymorphisms in the IL-6 gene and ischemic heart failure was found.

Relationship between polymorphisms in the TNF-α and IL-6 genes and IHD, as one of the most common causes of CHF, has been reported but remains controversial. Bennet *et al* reported no significant association between five SNPs in the TNF-α promoter region (-238G/A, -308G/A, -857C/T, -863C/A, and -1031T/C) and risk of Myocardial Infarction (MI)^27^. Similarly, allele frequencies, and genotype and haplotype distributions of the TNF-α promoter polymorphisms -863C/A and -308G/A were not related to the risk of CAD and MI^28^. In another study, none of the four TNF-α SNPs (-806C/T, -308G/A, -238G/A, and +467G/A) investigated reached statistical significance in the total sample of patients; however, a significant interaction between 238G/A polymorphism and risk of CHD was reported among nonsmokers in Chinese Han population^29^. Regarding the role of IL-6 gene polymorphisms in CHD, in a very recent meta-analysis, it was shown that the IL-6 -174G/C polymorphism is not significantly associated with increased risk of CHD; however, a significant association can be found between the -572- G/C polymorphism in the IL-6 gene and CHD risk, especially in Asian populations^30^.

While significant differences in some cytokine gene polymorphisms were found between the two groups of patients and controls, the small sample size can affect the robustness of the findings. Moreover, since the role of TNF-α and IL-6 SNPs in the pathogenesis of CHD has remained inconclusive, investigating patients with IHD and no heart failure might have given more conclusive results.

## Conclusion

In conclusion, this study demonstrates the association between certain allele, genotype, and haplotype frequencies in TNF-α gene with ischemic heart failure. Further investigation, using a larger sample size, to obtain more conclusive data regarding the role of TNF-α genotype in the pathogenesis of ischemic heart failure and influence on TNF-α level is warranted.
